# Systematic Characterization of AML Cell Lines to Support Preclinical In Vitro CAR-T Research

**DOI:** 10.3390/cells15211964

**Published:** 2026-10-28

**Authors:** Vassa D. Davydova, Anastasia A. Zotova, Nikolay M. Kapranov, Dmitry V. Dianov, Yulia O. Davydova, Tatiana A. Nenasheva, Irina Y. Zaichenko, Sergei D. Losev, Kseniia A. Nikiforova, Irina V. Galtseva, Apollinariya V. Bogolyubova

**Affiliations:** 1 Center for Precision Genetic Technologies for Medicine, National Medical Research Center for Hematology, Moscow 125167, Russia; davydova.vd@blood.ru (V.D.D.); zotova.aa@blood.ru (A.A.Z.); dianov.d@blood.ru (D.V.D.); nenasheva.ta@blood.ru (T.A.N.); zaichenko.i@blood.ru (I.Y.Z.); losev.sd@blood.ru (S.D.L.); 2 National Medical Research Center for Hematology, Moscow 125167, Russia; kapranov.n@blood.ru (N.M.K.); davydova.yuo@blood.ru (Y.O.D.); nikiforova.k@blood.ru (K.A.N.); galtseva.i@blood.ru (I.V.G.)

**Keywords:** acute myeloid leukemia, chimeric antigen receptor, in vitro CAR-T study, co-culture, cytotoxicity, AML cell line

## Abstract

**Highlights:**

**What are the main findings?**

- Commonly used AML cell lines may not recapitulate primary AML antigen profiles.
- Non-specific T cell-mediated killing may confound in vitro CAR-T efficacy assessment.

**What are the implications of the main findings?**

- We developed scoring framework integrated antigen expression and functional characteristics to rank AML cell lines.
- OCI-AML2, OCI-AML5, MV4-11, and MOLM-13 emerged as candidate models for clinically relevant in vitro CAR-T testing.

**Abstract:**

The development of chimeric antigen receptor (CAR) T cell therapies for acute myeloid leukemia (AML) remains challenging, underscoring the need for reliable preclinical models. Although AML cell lines are widely used in CAR-T studies, their ability to reflect clinically relevant antigen expression patterns and functional characteristics of primary AML cells has not been systematically evaluated. We characterized nine AML cell lines (KG-1, HL-60, Kasumi-1, OCI-AML2, OCI-AML5, THP-1, U-937, MV4-11, and MOLM-13) as targets for in vitro CAR-T studies. We assessed the expression of established CAR-T target antigens (e.g., CLL1, CD33), cell lines similarity to minimal residual disease (MRD) cells and matched healthy monocytes from 161 AML patients, the expression of emerging AML-associated antigens (Siglec-6, CSF1R, and CD70), growth behavior, and susceptibility to non-specific killing by non-transduced T cells. AML cell lines demonstrated substantial heterogeneity in antigen expression with several cell lines showing antigen densities exceeding those observed in AML blasts. Several widely used lines, including U-937, were susceptible to non-transduced T-cell cytotoxicity, potentially confounding CAR-T functional assays and overestimating CAR-T activity in vitro. The examined parameters were integrated into a scoring framework to rank AML cell lines according to their suitability for in vitro CAR-T studies. Based on this framework and additional assay-related considerations, OCI-AML2, OCI-AML5, MV4-11, and MOLM-13 were identified as most suitable for in vitro evaluation of CAR-T products, particularly those targeting CD33 and CLL1. These findings may facilitate more informed selection of AML cell lines for preclinical development of CAR-T therapies for AML.

## 1. Introduction

Acute myeloid leukemia (AML) is the most common acute leukemia in adults, accounting for approximately 80% of cases, and it remains associated with poor clinical outcomes, particularly in patients older than 65 years [1,2,3]. Although intensive chemotherapy and allogeneic hematopoietic stem cell transplantation (allo-HSCT) remain the standard of care, treatment outcomes are limited by frequent relapse and significant treatment-related toxicity [4]. The use of high-dose chemotherapy may also be limited for frail patients or those with comorbidities [5]. Furthermore, more than 30% of patients with AML relapse after allo-HSCT, and the five-year overall survival rate remains between 30% and 50% [6]. The high recurrence rate and relatively low survival rates in patients with AML underscore the need to identify new and improve existing therapy strategies. 

Chimeric antigen receptor (CAR) T therapy is one of the rapidly evolving and promising areas among new therapeutic approaches for AML [7,8]. However, its preclinical development relies heavily on in vitro cytotoxicity assays, in which CAR-T cells are tested against tumor cell lines expressing the target antigen [9,10,11,12,13,14,15]. A critical but often underappreciated challenge in this setting is the choice of the target AML cell line used for the evaluation of CAR products. In many studies, model cell lines are selected primarily based on target antigen expression, without systematic consideration of their broader immunophenotypic profile, growth kinetics, or intrinsic sensitivity to primary activated T cells. However, these factors can profoundly influence cytotoxicity readouts. For example, primary non-transduced T cells, routinely used as negative controls, may exert substantial background killing through CAR-independent pathways such as Fas-FasL-mediated cytotoxicity, and highly vulnerable tumor lines can exaggerate apparent CAR-T efficacy [16,17]. These considerations are particularly relevant for the interpretation of subsequent preclinical studies, as different AML models are sometimes used for in vitro functional testing and in vivo validation [18]. AML cell lines differ substantially in their immunophenotypic, molecular, and functional characteristics and should therefore not be considered interchangeable models [19]. Although the use of multiple models is valuable for preclinical evaluation, differences between target models may complicate the assessment of in vitro-to-in vivo concordance [15]. These considerations support the need for a systematic, multiparametric approach to AML cell-line selection for CAR-T testing.

The aim of this study was to perform a comparative characterization of a panel of nine AML cell lines, including KG-1, HL-60, Kasumi-1, OCI-AML2, OCI-AML5, THP-1, U-937, MV4-11, and MOLM-13, to identify the most relevant preclinical models for in vitro CAR-T testing, considering target antigen expression, complex immunophenotype, proliferation and viability under assay conditions, and baseline susceptibility to non-specific T cell-mediated cytotoxicity.

## 2. Materials and Methods

### 2.1. Patients and Samples

This study included bone marrow samples from 161 patients with AML who were considered a treatment-failure cohort and potential candidates for CAR-T cell therapy and peripheral blood samples from 3 newly diagnosed AML patients. Peripheral blood samples were also collected from 6 healthy donors for generation of CAR-T cell products and for the assessment of antigen expression on healthy donor monocytes. Studies involving human participants were reviewed and approved by the Research Ethics Committee of the National Medical Research Center for Hematology (protocol no. 87, 17 February 2025).

Patients underwent routine clinical minimal residual disease (MRD) testing in the immunophenotyping laboratory at the National Medical Research Center for Hematology, Moscow, Russian Federation. According to recent European LeukemiaNet (ELN) recommendations [20], MRD relapse is defined as MRD ≥ 0.1%, and indicates a high probability of treatment failure. In the present study, we applied a stricter threshold of MRD ≥ 1% to select patients with more evident signs of treatment failure and to ensure that a sufficient number of leukemic cells were available for robust assessment of median fluorescence intensity (MFI). Included patients did not receive any surface antigen-targeted treatment. Since our goal was to compare immunophenotype profiles of patients’ AML cells after non-targeted therapy, we did not stratify the cohort by number of therapy lines, allo-HSCT status, cytogenetic or molecular subgroups, or French–American–British (FAB) classification. The median at MRD testing was 51 years (range, 14–87). MRD levels varied from 1.01% to 96.8%, with a median of 9.24% among all nucleated bone marrow cells.

For the evaluation of emerging AML-associated antigens, peripheral blood samples were collected prior to initiation of therapy and used for flow cytometric assessment of antigen expression on circulating leukemic blasts.

### 2.2. Cell Line Detection and Authentication, and Cell Culture

A panel of nine established AML cell lines was tested: KG-1, HL-60, Kasumi-1, OCI-AML2, OCI-AML5, THP-1, U-937, MV4-11, and MOLM-13. All the cell lines except for OCI-AML2, OCI-AML5, MOLM-13 were obtained from ATCC. OCI-AML2, OCI-AML5, MOLM-13 were obtained from DSMZ. To ensure identity and minimize cross-contamination, each cell line stock was routinely tested for mycoplasma contamination (MycoReport, Evrogen, Moscow, Russia) and authenticated by short-tandem repeat (STR) profiling using a standard human STR-locus panel (health genetech express g28 (wt); GTZ, Moscow, Russia). For each AML cell line, baseline characteristics were compiled from the original cell line documentation and the peer-reviewed literature [19]. These characteristics include the cell line origin, cytogenetic background, and immunophenotype.

Cell lines were cultured in IMDM (Thermo Fisher Scientific, Waltham, MA, USA) medium supplemented with 10% fetal bovine serum (FBS) (Biowest, Nuaillé, France). Penicillin-streptomycin was added to the culture medium at a final concentration 5 U/mL penicillin and 5 µg/mL streptomycin (Paneco, Moscow, Russia). All cell lines were passaged at cell densities of 0.5–1  ×  10^6^ cells/mL and incubated under standard conditions (37 °C, 5% CO_2_).

### 2.3. Isolation and Cell Culture of Primary Immune Cells

Peripheral blood mononuclear cells (PBMCs) were isolated from healthy donor peripheral blood by density gradient centrifugation using Ficoll (1.077 g/cm^3^; Paneco, Moscow, Russia). CD3+ T cells were enriched using magnetic separation with CytoSinct CD3 nanobeads (GenScript, Piscataway, NJ, USA) on magnetic columns, according to the manufacturer’s instructions. Purified T cells were activated using anti-CD3/CD28 Enceed T cell activation nanobeads (GenScript, Piscataway, NJ, USA) and cultured in ImmunoCult™-XF T Cell Expansion medium (StemCell Technologies, Vancouver, BC, Canada), supplemented with recombinant human IL-2 (50 U/mL) (Biotech, Saint Petersburg, Russia) and 5 U/mL penicillin and 5 µg/mL streptomycin.

### 2.4. Isolation and Cell Culture of Human Primary AML Blasts 

Primary AML blasts were obtained from leukapheresis of 3 patients with peripheral blood blast counts exceeding 81% (range 81–97%). Cells were washed twice with phosphate-buffered saline (PBS) containing 2 mM EDTA (SERVA Electrophoresis GmbH, Heidelberg, Germany) to remove residual plasma. Cell pellets were resuspended and maintained in IMDM supplemented with 10% FBS under standard culture conditions. Penicillin-streptomycin was added to the culture medium.

### 2.5. In Vitro Cell Viability Assays

To ensure comparable conditions, all cell lines were thawed simultaneously and passaged 3–4 times before being included in the experiments. Cells were seeded at an initial density of 2–3 × 10^5^ cells/mL, corresponding to the lower limit of the optimal cell density range for all cell lines, in three different culture conditions: IMDM medium supplemented with 10% FBS, ImmunoCult medium with/without recombinant human IL-2 (50 U/mL). Cells were maintained for 7 days without medium replacement to assess the impact of culture conditions on survival. Cell viability was evaluated by flow cytometry using 7-aminoactinomycin D (7-AAD; BioLegend, San Diego, CA, USA) staining without washing steps using MACS Quant 10 volumetric cytometer (Miltenyi Biotec, Bergisch Gladbach, Germany). Population doubling time was calculated from the exponential growth phase using the formula $T=\frac{\ln(2)\times T}{\ln\frac{N}{N0}}$, where T is the time interval, N and N0 are the cell numbers at the end and beginning of the exponential phase, respectively.

### 2.6. Surface Marker Assessment by Flow Cytometry

Routine clinical MRD monitoring was performed on 161 bone marrow samples. MRD analysis was based on the “different from normal” approach [20], supplemented by leukemia-associated aberrant immunophenotype (LAIP) information. Samples were processed using a bulk-lysis approach to concentrate white blood cells, followed by incubation with monoclonal antibodies, washing, and acquisition on DxFLEX flow cytometer (Beckman Coulter, Brea, CA, USA), and the resulting data were processed using Kaluza (version 2.3, Beckman Coulter, Brea, CA, USA) or FlowJo software (version 10.1, FlowJo, LLC, Ashland, OR, USA).

Samples were stained with the fluorochrome-conjugated anti-human monoclonal antibodies (mAb) listed in Table S1 (MRD panel). When available, LAIP-associated markers included CD2, CD4, CD5, CD7, CD19, or CD56 depending on the diagnostic immunophenotype and their utility for MRD assessment; CD11a was used in other cases. In 161 samples, MRD cells and non-aberrant monocytes were gated. MFI of MRD AML cells and monocytes were extracted for subsequent analysis together with percentage of MRD cells expressing individual antigens. All cell lines were thawed simultaneously and passaged 3–4 times before staining. For visualization purposes, AML cell lines were mixed with peripheral blood (PB) cells from a healthy donor at a 20:80 ratio. AML cell lines and cell line-PB mixtures were stained with the same mAb panel with CD7 as a LAIP-based antigen. We analyzed MFI of each marker and the percentage of cells expressing each antigen. Monocytes were identified as CD14^+^ cells (antiCD14, clone TÜK4, #130-113-153, Miltenyi Biotec, Bergisch Gladbach, Germany). Antibodies targeting emerging antigens are listed in Supplementary Table S2 (Additional markers). Unstained cells from each line were used as a negative control.

### 2.7. Co-Culture Assays

Co-culture experiments were performed by incubating tumor cell lines with primary activated T cells from healthy donors at defined effector-to-target (E:T) ratios (5:1, 1:2, 1:4). Cells were seeded with final density of 1 × 10^6^ cells/mL and cultured in ImmunoCult medium with IL-2 (50 U/mL), under standard conditions for 5 days. At days 0, 2 and 5, co-cultures were stained with 7-AAD to distinguish live and dead cells without washing steps using MACS Quant 10 volumetric cytometer. Target AML cell lines were previously engineered to express NanoLuc and GFP, allowing quantification of residual target cells based on GFP expression by flow cytometry. The percentage reduction in viable target cell number (L) following co-culture of AML cell lines with primary non-transduced T cells was calculated using the formula L = 100 − (n/m × 100), where n is the number of GFP+ target cells at day 2 or 5, and m is the number of GFP+ target cells at day 0. IFN-γ production was quantified using the Gamma-Interferon-EIA-BEST ELISA kit (Vector-Best, Novosibirsk, Russia) according to the manufacturer’s instructions.

The surface expression of Fas (CD95) (APC, clone DX2, BioLegend, San Diego, CA, USA, #305612), and HLA-ABC (APC, clone W6/32, BioLegend, #311410) on cell lines were assessed by flow cytometry. As isotype control anti-CD8 (APC, clone SK1, Becton Dickinson, San Jose, CA, USA, #340659) was used. 

### 2.8. Principal Component Analysis (PCA)

To simplify visualization of flow cytometry data acquired from 161 AML MRD samples we performed PCA. MFI values of 15 studied antigens (CD15, CD34, CD117, CD33, CD13, CD14, CD11b, HLA-DR, CD45, CD38, CD371, CD99, CD123, CD45RA, and CD10) were included in the analysis. Collected data were transformed by adding a constant value to each data point so that MFI was over 1 and then applying the base-10 logarithm. Normality of data distribution was tested by Shapiro–Wilk tests. Data were scaled based on medians and interquartile intervals using RobustScalerfunction from the Python library to exclude high impact of outliers and non-normality of distribution. The calculated median and interquartile intervals from this analysis were stored for transformation of the remaining data. Finally, PCA was trained and applied to the transformed data. The described transformation and analysis pipeline was applied to MFI data from non-MRD monocytes and cell lines.

### 2.9. Literature Analysis

For the literature analysis, each AML cell line was searched individually in the PubMed database using predefined combinations of key terms related to CAR-T therapy and experimental setting. For in vitro studies, search terms included cell line names, CAR-T-related terms (“CAR-T” OR “CAR T” OR “chimeric antigen receptor” OR “CAR”) combined with “in vitro”, “co-culture”, “cytotoxicity”, “killing”, or “cytokine release” and “leukemia”, “AML” or “acute myeloid leukemia” terms. For in vivo studies, cell line names, CAR-T-related terms, and “leukemia”, “AML” or “acute myeloid leukemia” terms were combined with “in vivo”, “xenograft”, “mouse”, “murine”, or “engraftment” terms. The frequency of each AML cell line reported in preclinical CAR-T studies was summarized (Table S3).

### 2.10. Data Analysis and Statistical Methods

All statistical analyses were performed in R (version 4.5.2). Data analysis was performed using the dplyr (version 1.2.1) and tidyr (version 1.3.2) packages. Kruskal–Wallis tests, Friedman tests, pairwise Mann–Whitney tests, two-way ANOVA, and Tukey’s HSD tests were performed using base R functions. Linear mixed-effects models were built using lmerTest package (version 3.2-1). Assumptions for ANOVA testing were assessed using Levene’s test for homogeneity of variance (car (version 3.1-5) package) and the Shapiro–Wilk test for normality of residuals (base R functions). Estimated marginal means (EMMs) were calculated using the emmeans (version 2.0.4) package. Area under the curve (AUC) scores were calculated with the DescTools (version 0.99.60) package. Differences in viable target cell reduction among AML cell lines at each E:T ratio were assessed by a Friedman test followed by pairwise Wilcoxon post hoc tests for paired measurements, with Benjamini–Hochberg correction for multiple comparisons (FDR control). Doubling times and AUC scores were each analyzed by two-way ANOVA (Cell line × Culture Media) in order to assess the effect of culture media on AML cell lines’ growth rate. Cell line expansion at different E:T ratios was assessed using linear mixed-effects model with Donor specified as a random intercept. Pairwise comparisons between cell lines within each medium were performed using Tukey’s HSD-adjusted comparisons of estimated marginal means (EMMs). The data were visualized using the GraphPad Prism software, version 10.5.

## 3. Results

### 3.1. Evaluating AML Cell Lines Based on Clinical Relevance and CAR-T Target Antigen Expression

Current preclinical drug-development studies often employ AML cell lines as models for in vitro and in vivo experiments. We evaluated the clinical relevance of a panel of nine widely used AML cell lines, including KG-1, HL-60, Kasumi-1, OCI-AML2, OCI-AML5, THP-1, U-937, MV4-11, and MOLM-13, by measuring parameters relevant to preclinical workflows, including immunophenotyping, growth kinetics, and cytotoxicity assays routinely applied in CAR-T therapy development. Baseline characteristics of these cell lines, including FAB subtype, origin, cytogenetics, and lineage markers, are summarized in Table 1.

**Figure 1:**
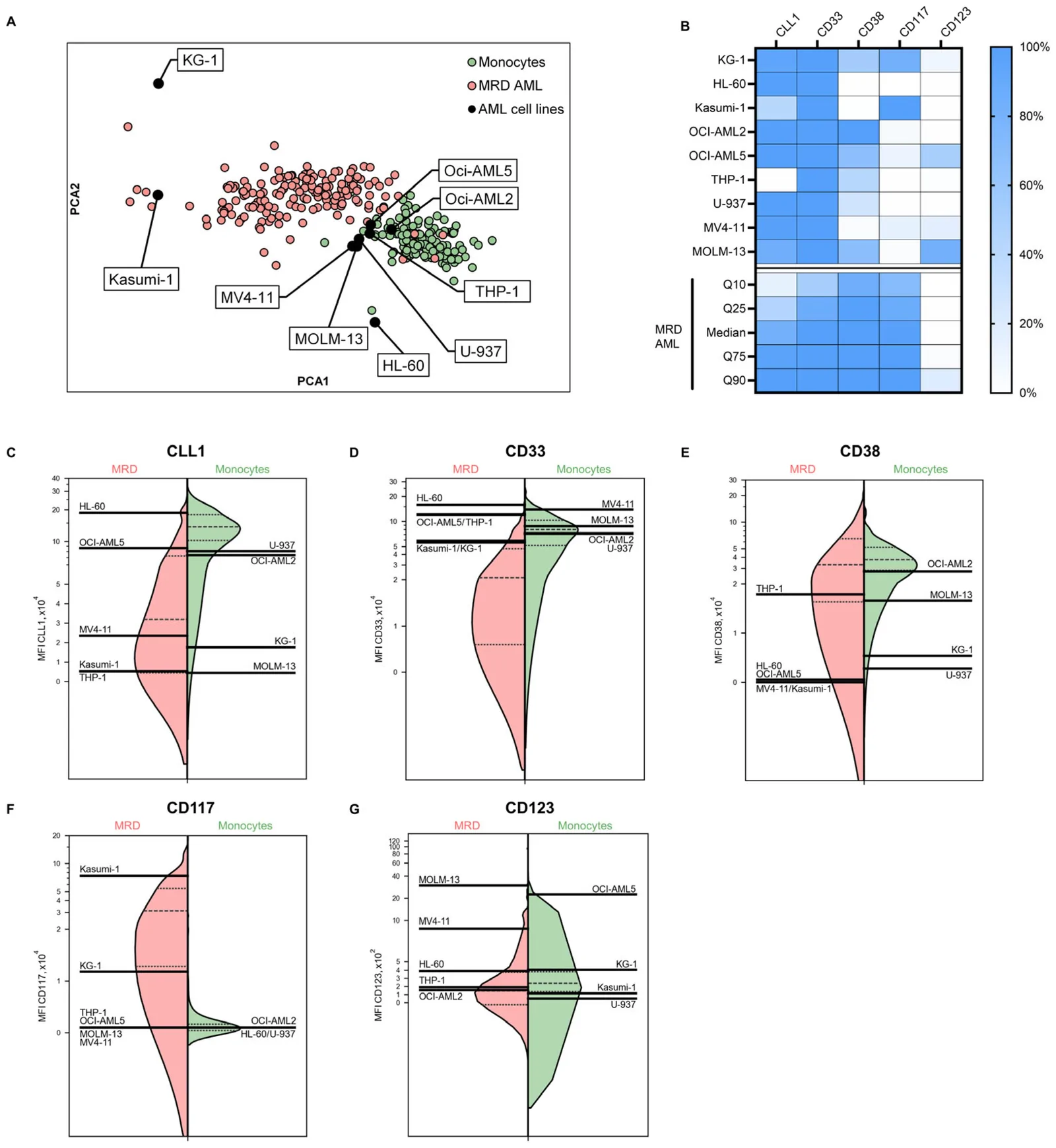
Integrated immunophenotypic comparison of AML cell lines with patient-derived MRD blasts and monocytes. (**A**) Principal component analysis (PCA) of flow cytometric marker expression in AML cell lines, patient-derived AML MRD blasts, and monocytes. (**B**) Heatmap showing the percentage of antigen-positive cells (%) for five AML-associated antigens across AML cell lines. Values shown below each antigen represent the 10th, 25th, 50th (median), 75th, and 90th percentiles of the percentage of antigen-positive cells in the patient-derived AML MRD blast cohort. (**C**–**G**) Violin plots showing the expression of major AML-associated markers (CLL1, CD33, CD38, CD117, CD123) in patient-derived AML MRD blasts and healthy monocytes, with median fluorescence intensity (MFI) of AML cell lines indicated by horizontal lines. The thick hatching indicates the median, whereas the fine hatching indicates the 25th and 75th percentiles.

To address how closely these AML cell lines resemble primary AML blasts and patients’ healthy monocytes, we performed PCA of cytometry-derived marker expression profiles (Figure 1A, Table S1), based on MFI values of surface markers routinely used for MRD detection in AML. Leukemic blasts obtained from 161 patients with AML formed a dispersed cluster with considerable within-group heterogeneity, consistent with inter-patient variability in AML immunophenotype. Matched healthy monocytes localized adjacent to the patient-blast cluster were partially overlapping. Most AML cell lines (OCI-AML2, OCI-AML5, MOLM-13, THP-1, MV4-11, U-937) were positioned at the interface between the blast and monocyte clusters, reflecting intermediate immunophenotypic features. The Kasumi-1 occupied the patient blasts-peripheral edge, distant from the monocyte cluster center, whereas KG-1 and HL-60 were located far from patient blasts, indicating divergent immunophenotypes. Collectively, these findings demonstrate that AML cell lines displayed considerable immunophenotypic heterogeneity.

We next assessed the proportion of antigen-positive cells across AML cell lines and MRD samples for relevant CAR-T antigens including CLL1, CD33, CD38, CD117, and CD123 (Figure 1B). The selected antigens have been well documented to be expressed on AML blasts and have been investigated as potential therapeutic targets in AML [8]. Expression patterns ranged from uniformly high (>99% CD33+ cells) to heterogeneous across analyzed antigens. The CLL1 antigen was expressed by most cell lines (84–100%), with the exception of Kasumi-1 (41%) and THP-1 (0.5%), which fell below the first quartile of patient MRD samples (median = 82%). CD38 showed marked variability with OCI-AML2 being uniformly positive (100%), whereas OCI-AML5, KG-1, MOLM-13, THP-1, and U-937 showed intermediate positivity (27–68%). All remaining cell lines had < 5% CD38-positive cells. CD117 was predominantly expressed by Kasumi-1 (99.9%) and KG-1 (82%), whereas CD123 showed generally low or negligible positivity across cell lines and MRD samples with the exception of OCI-AML5 and MOLM-13.

To further assess the clinical relevance of the analyzed cell lines, violin plots were used for side-by-side comparison of antigen expression in the cell line panel, patients’ MRD cells and healthy monocytes (Figure 1C–G). Given that normal monocytes may represent a major site of on-target/off-leukemia toxicity following CAR-T therapy targeting myeloid antigens, we included this population alongside MRD blasts in our analysis. Importantly, for most antigens, the median MFI values were higher in healthy monocytes than in MRD cells, except for the CD117 antigen. This observation indicates well-known risks of on-target/off-leukemia recognition and subsequent toxicity [27,28]. Individual cell lines occupied distinct positions within these reference distributions. CLL1, MV4-11, Kasumi-1, THP-1, KG-1, and MOLM-13 showed expression below the MRD median, while the remaining cell lines exceeded the upper quartile and approached the monocyte range. HL-60 showed the highest CLL1 expression, exceeding the upper quartile of healthy monocytes. In contrast, CD33 expression consistently resembled healthy monocytes and exceeded the MRD upper quartile in all cell lines. CD38, OCI-AML2, THP-1, and MOLM-13 fell within the MRD interquartile range, whereas all other cell lines expressed lower antigen levels. CD117 expression was generally low, except for KG-1 and Kasumi-1, while CD123 largely resembled MRD distribution but was increased in MOLM-13, OCI-AML5, and MV4-11. Expression patterns of additional MRD markers are shown in Figure S1A–I. We also observed autofluorescence in HL-60 and THP-1 (Figure S1J,K) with cell line MOLM-13 shown for comparison in the corresponding channels (Figure S1L,M), a feature that should be considered when fluorescent reporters are used to quantify CAR-T-mediated cytotoxicity.

To determine whether AML cell lines could be reliably identified within a heterogeneous cellular background, AML cell lines were mixed with healthy donor PBMCs at 20:80 ratio, and analyzed using MRD flow cytometry panel (Figure S2). In mixed cultures, only THP-1, MV4-11, and MOLM-13 remained consistently distinguishable from PBMC subsets across multiple markers. CD117 enabled clearer discrimination, whereas CLL1, CD33, and CD123 partially overlapped with monocytes, reflecting shared antigen expression between malignant and normal myeloid cells.

Taken together, several AML cell lines exhibited antigen densities exceeding that of leukemic MRD cells—including all analyzed cell lines for CD33 and cell lines HL-60 and OCI-AML5 for antigen CLL1. While such high antigen relative expression may increase the apparent sensitivity of CAR-T cells in vitro, it may also overestimate therapeutic efficacy and fail to accurately reflect the antigen landscape encountered in patients.

### 3.2. Expression of Emerging AML-Associated Antigens Relevant for CAR-T Cell Targeting

**Figure 2:**
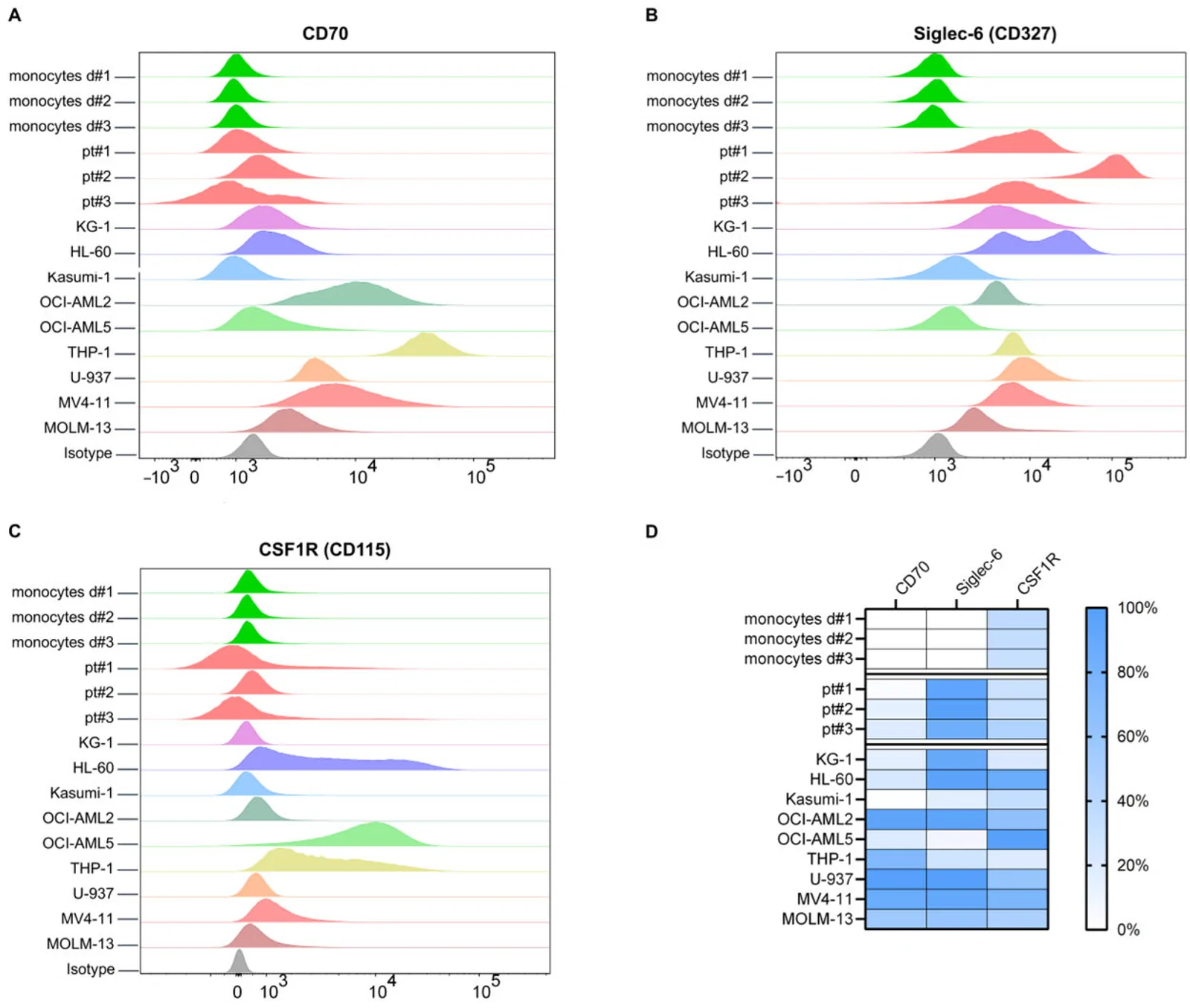
Comparative flow cytometric profiling of emerging AML targets in cell lines, patient-derived blasts, and monocytes from healthy donors. (**A**–**C**) Flow cytometry histograms showing the expression of emerging AML targets (CD70, Siglec-6, CSF1R) in AML cell lines, three patient-derived AML blast samples isolated from peripheral blood, and monocytes from healthy donors**.** Antigen expression was quantified as MFI. MFI distributions across individual samples are shown as stacked panels. (**D**) Heatmap summarizing antigen expression patterns based on the percentage of antigen-positive cells across AML cell lines, patient-derived AML blasts, and healthy-donor monocytes.

To extend the immunophenotypic characterization of the AML cell lines beyond conventionally assessed MRD markers, we next evaluated three emerging CAR-T target antigens: Siglec-6 (CD327), CSF1R (CD115), and CD70. These targets are being actively explored by multiple research groups: CAR-T cells directed against Siglec-6 (NCT05488132) and CD70 (NCT04662294, NCT06326463) have already entered clinical trials, whereas CAR-T cell products directed against CSF1R remain in the preclinical development stage [29]. These markers were not included in the standardized MRD flow cytometry panel; therefore, this analysis was performed in comparison with an independent cohort of diagnostic peripheral blood samples obtained from three AML patients with abundant circulating blasts (>81%) in peripheral blood. Antigen expression was further compared with that of healthy donor monocytes to provide an assessment of expression on normal myeloid cells (Figure 2).

Primary patient AML cells exhibited variable but generally high Siglec-6 expression, while the Kasumi-1, OCI-AML5 and MOLM-13 showed low-level expression and all other cell lines were Siglec-6-positive. Siglec-6 staining was not observed on healthy monocytes. CD70 expression on patient blasts was uniformly low, albeit higher than monocytic CD70; among cell lines, CD70 was high on THP-1, low on HL-60, KG-1 and OCI-AML-5, and absent on Kasumi-1. In contrast, CSF1R was detectable on all cell lines and on AML blasts, with expression levels comparable to that of healthy monocytes, and was most strongly expressed on OCI-AML5. The characteristic bimodal staining profile observed with the HL-60 cell line may be explained by high levels of cellular autofluorescence (see Figure S1K). Taken together, these data indicate that CSF1R is widely expressed in both cell lines and primary AML blasts, whereas Siglec-6 and CD70 exhibit a more limited and heterogeneous expression profile.

### 3.3. Assessment of Proliferation and Viability of AML Cell Lines Under Different Conditions 

**Figure 3:**
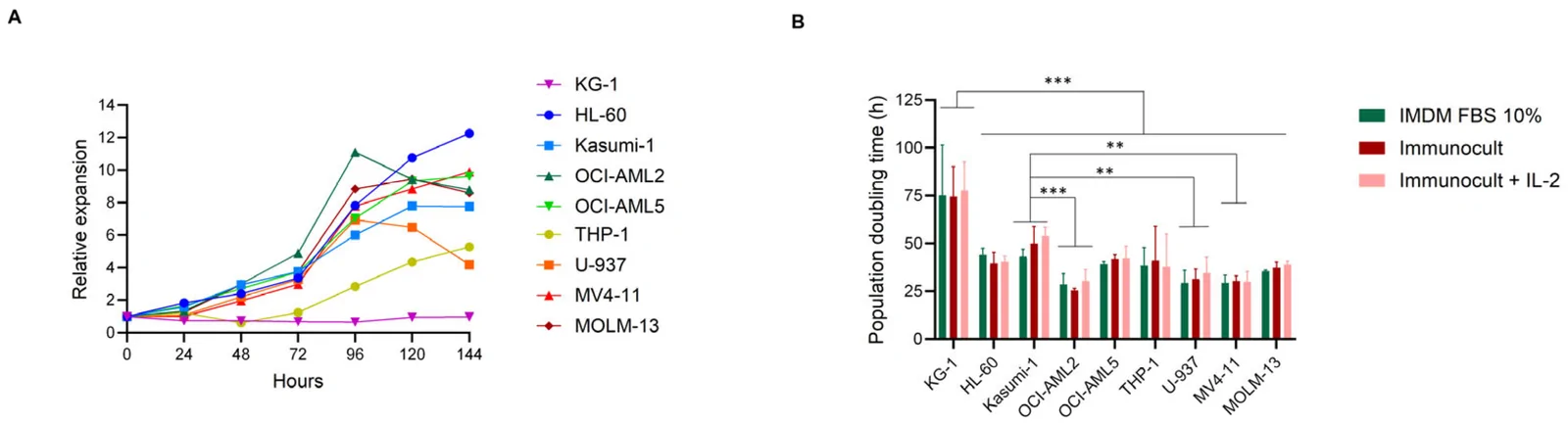
Assessment of proliferation and viability of AML cell lines under different culture conditions. AML cell lines were cultured in IMDM supplemented with 10% FBS, ImmunoCult, or ImmunoCult supplemented with IL-2. Cell proliferation was assessed by serial cell counting over time. (**A**) Representative growth curves of AML cell lines cultured in ImmunoCult supplemented with IL-2, showing cell expansion over time. (**B**) Doubling time of each AML cell line under the three tested culture conditions. Data are presented as mean ± SD. Two-way ANOVA (Cell Line × Culture Medium) revealed that the culture medium had no significant effect on the population doubling time for any of the AML cell lines evaluated; this factor (culture medium) was omitted from subsequent analysis, allowing for a direct comparison of cell lines using Tukey’s HSD test. ** *p* < 0.01, *** *p* < 0.001.

To evaluate the growth characteristics of AML cell lines as an important determinant of their suitability for cytotoxicity assays, the cell lines were cultured in three different media: a standard maintenance medium (IMDM supplemented with 10% FBS), and two variants of the serum-free medium commonly used in CAR-T production and cytotoxicity assays (ImmunoCult™-XF T Cell Expansion medium, StemCell Technologies, Vancouver, BC, Canada), with and without adding IL-2. This approach allowed the assessment of both baseline proliferation under standard conditions and the ability of each line to adapt to the media used in subsequent cytotoxicity assays. Growth kinetics and viability dynamics were monitored over 168 h (7 days), with cell counts performed every 24 h (Figure 3).

Among all models, KG-1 exhibited the longest population doubling time and the most unstable growth: its maximal cell density did not reach 1 × 10^6^ cells/mL by the end of the experiment in any of the tested media, and this cell line was therefore considered unsuitable for subsequent functional assays (Figure 3). U-937 showed the poorest adaptation to ImmunoCult media, with a lower peak cell density than in the standard media and a pronounced decline in viability after 120 h of culture (Figure S3G). Kasumi-1 also adapted significantly worse to the ImmunoCult medium transfer, although no sudden cell death was observed (Figure S3C). For the remaining cell lines, no significant differences in baseline viability were observed between the different culture conditions (Figure S3). Differences in adaptation to culture conditions and doubling time may reflect intrinsic biological differences between individual AML cell lines, including their cell of origin.

### 3.4. Susceptibility of AML Cell Lines to Non-Specific T Cell Cytotoxicity

A major challenge in in vitro CAR-T cytotoxicity assays is distinguishing CAR-mediated target cell killing from the intrinsic cytotoxic activity of non-transduced T cells (NT). NT cells are usually considered as a negative control in co-culture experiments, as they lack antigen-specific effector potential [30,31]. Nevertheless, they can still cause substantial cytotoxicity through bystander mechanisms that do not require CAR engagement or direct antigen recognition [16,17,32].

**Figure 4:**
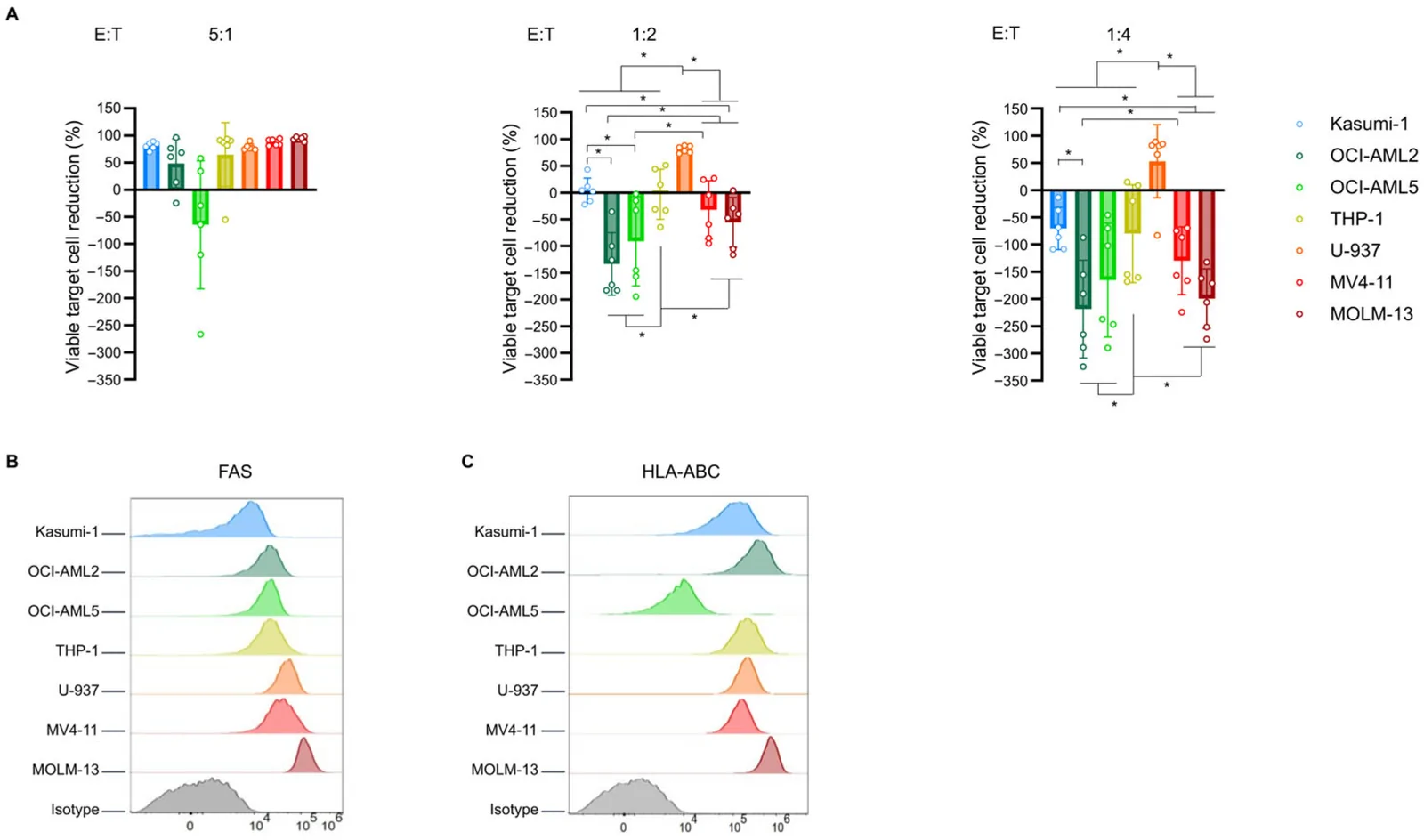
Susceptibility to non-specific T cell cytotoxicity and expression of Fas and HLA class I in AML cell lines. (**A**) Percentage reduction in viable target cell number following co-culture of AML cell lines with primary non-transduced T cells from six donors at effector-to-target (E:T) ratios of 5:1, 1:2, and 1:4 (*n* = 6 biological replicates). Each AML cell line was tested with T cells from all donors. The day-0-normalized reduction in viable target cell number reflects the balance between AML cell growth and T-cell-mediated target cell killing. Negative values indicate an increase in viable target cell number despite T-cell exposure. For each E:T ratio, differences in viable target cell reduction among AML cell lines were assessed using the Friedman test followed by pairwise Wilcoxon signed-rank tests. *p* values were adjusted for multiple comparisons using the Benjamini–Hochberg method to control the false discovery rate (FDR). Data are presented as mean ± SD. * *p* < 0.05. (**B**) Representative flow cytometry histograms showing cell-surface Fas expression on AML cell lines compared with isotype controls. (**C**) Representative flow cytometry histograms showing cell-surface HLA-ABC expression on AML cell lines compared with isotype controls.

To address this challenge, we evaluated changes in target cell number during co-culture with NT cells. NT cells were activated with CD3/CD28 beads and IL-2, and co-cultured with AML cell lines at three effector-to-target (E:T) ratios (Figure 4A, Figure S4). In these assays, target cell sensitivity to T cell-mediated cell death was observed across all cell lines and increased proportionally with NT cell density. Among them, U-937 demonstrated markedly greater reduction in viable target cell number during co-culture with NT cells. In contrast, OCI-AML5 and OCI-AML2 were less sensitive to cytotoxic activity of NT cells. This finding indicates that AML models can be highly sensitive to non-specific T cell-mediated killing, even in the absence of CAR expression. IFN-γ production in co-cultures reached the peak by day 2, ranging from 500 to 1700 pg/mL (Figure S4H). While no statistically significant differences in IFN-γ production were observed across the tested AML cell lines, co-cultures with cell lines U-937 and Kasumi-1 consistently exhibited the highest IFN-γ levels, whereas co-cultures with cell lines OCI-AML2 and OCI-AML showed the lowest concentrations.

To explore the potential mechanisms of CAR-independent reduction in viable target cell number, we proposed that the cell lines were sensitive to T cell-mediated cytotoxicity and thus their baseline surface expression of Fas (CD95) was quantified (Figure 4B). Despite heterogeneous Fas expression across the panel, we did not find a correlation between Fas levels and the reduction in target cell number during NT cell co-culture, indicating that Fas expression alone does not explain the observed differences between cell lines.

To further characterize potential factors associated with background T-cell cytotoxicity, we also quantified HLA class I (HLA-ABC) surface expression across the AML cell line panel (Figure 4C). Since our experimental system is inherently HLA-mismatched (donor-derived T cells and AML cell lines) we reasoned that differences in HLA class I expression may be relevant to allogeneic T-cell recognition and may contribute to CAR-independent cytotoxicity [33]. Notably, OCI-AML5 exhibited the lowest surface HLA-I expression in our panel.

### 3.5. Framework for Selecting Tumor Cell Lines for CAR-T Studies

**Figure 5:**
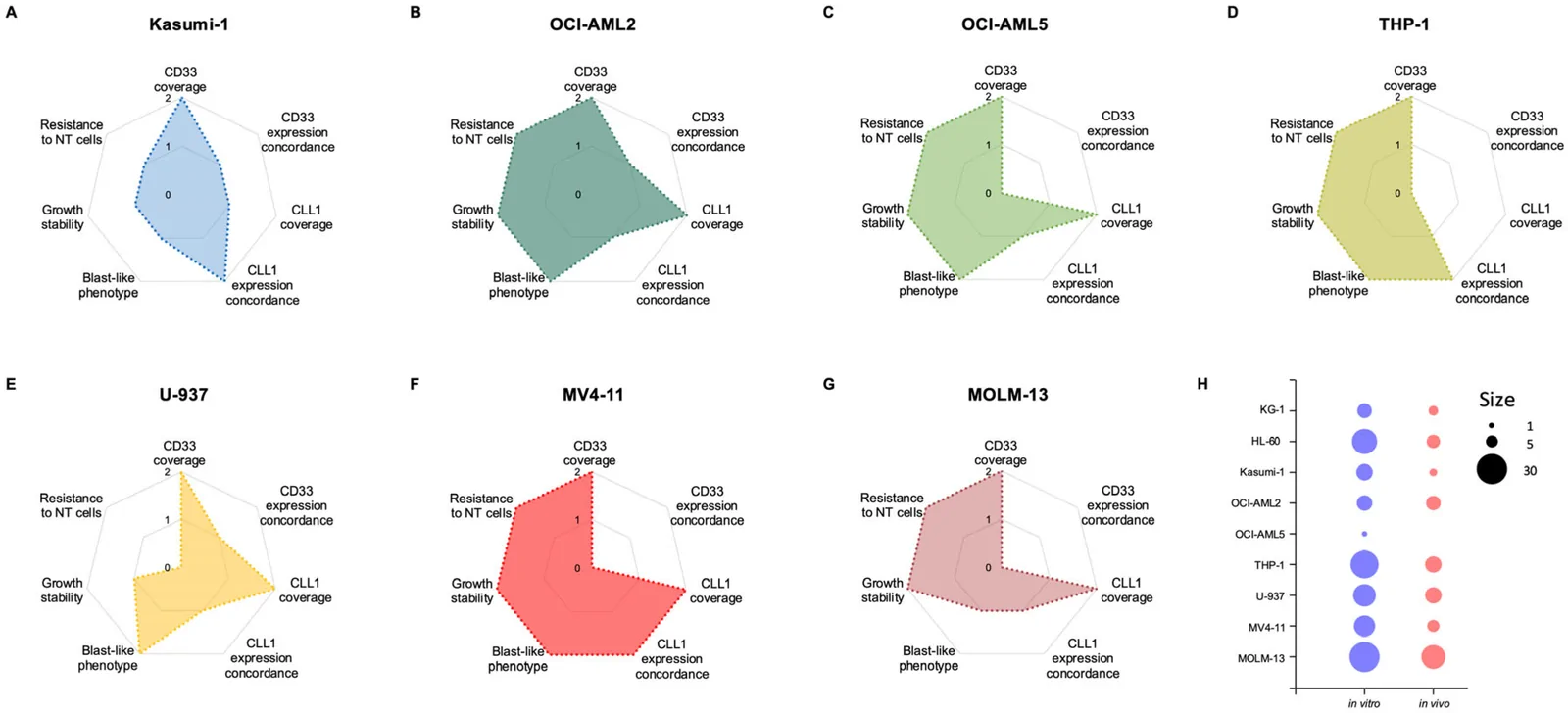
Selection framework for AML cell line models. (**A**–**G**) Radar plots showing the evaluation of AML cell lines according to seven predefined criteria: CD33 antigen coverage, CD33 antigen expression concordance, CLL1 antigen coverage, CLL1 antigen expression concordance, blast-like phenotype, growth stability, and resistance to T cell-mediated killing. Each criterion was scored on a 0–2 scale, with higher scores indicating greater suitability as an AML model (see Table S2). (**H**) Frequency of use of individual AML cell lines in reported in vitro and in vivo CAR-T cell studies (see Table S3), shown as a dot plot. Dot size represents the number of studies in which each cell line was used.

To facilitate a systematic comparison of AML cell lines, we developed a scoring system integrating seven parameters, enabling rapid assessment of the strengths and limitations of each model. Each parameter was evaluated on a three-point scale (0–2), with higher scores indicating greater suitability (Table 2, Figure 5A–G, Table S2). We selected CD33 and CLL1 as clinically relevant AML CAR-T targets, as both antigens have been extensively investigated in registered clinical trials, representing the most frequently explored targets for AML-directed CAR-T therapy. These antigens were evaluated via target coverage and antigen relative expression. We also included blast-like phenotype, growth stability, and T cell resistance as evaluation criteria for each cell line. Application of T cell resistance parameter was not feasible for two cell lines. HL-60 could not be reliably analyzed because of pronounced autofluorescence, while KG-1 exhibited insufficient proliferation to support co-culture experiments. Consequently, both cell lines were not included in the final integrated scoring analysis. Nevertheless, KG-1 and HL-60 were evaluated for all remaining parameters, and the corresponding scores are provided in Table S2.

According to the integrated scoring system, five cell lines (OCI-AML2, OCI-AML5, MV4-11, MOLM-13, and THP-1) achieved the highest overall scores. However, THP-1 exhibited pronounced autofluorescence (Figure S1J), which may interfere with fluorescence-based readouts commonly used in in vitro CAR-T assays. Therefore, despite its high overall score, THP-1 was excluded from the final selection, leaving four cell lines that may be considered suitable for in vitro CAR-T studies, with particular relevance to CLL1- and CD33-directed CAR-T products: OCI-AML2, OCI-AML5, MV4-11, and MOLM-13.

Importantly, the score reflects overall suitability as a CAR-T preclinical model and may not capture the specific requirements of individual experimental applications. Antigen-high models may be preferable for proof-of-concept experiments (e.g., OCI-AML2 for CLL1-directed CAR-T), whereas antigen-low or heterogeneous models may be more informative for assessing CAR-T sensitivity under limited or variable target expression (e.g., Kasumi-1 or THP-1 for CLL-1-directed CAR-T). Thus, model selection should be guided by the CAR target and specific experimental question, while the scoring system can help prioritize suitable models.

To assess current practice in AML CAR-T research, we also examined how frequently AML cell lines were used in published in vitro and in vivo CAR-T studies (Figure 5H, Table S4). Use of AML cell lines in preclinical CAR-T studies was markedly heterogeneous. HL-60, MV4-11, OCI-AML2, and MOLM-13 were among the most frequently used both in vitro and in vivo experiments, whereas OCI-AML5 was only rarely employed in in vitro studies despite its favorable performance across the majority of evaluated parameters based on our scoring system.

## 4. Discussion

In the present study, we systematically evaluated nine AML cell lines as preclinical models for CAR-T development by phenotypic similarity to patient samples, and functional parameters relevant for CAR-T assays. Based on the integrated scoring system and additional assay-related considerations, the cell lines OCI-AML2, OCI-AML5, MV4-11, and MOLM-13 were identified as the most suitable preclinical models for in vitro studies of CAR-T products, particularly those targeting CD33 and CLL1.

Our PCA revealed that AML cell lines exhibit considerable immunophenotypic heterogeneity. While most cell lines align with the distribution observed for patient-derived blasts and matched monocytes, KG-1, HL-60 display markedly divergent profiles, localizing far from patients’ MRD cells. Unlike patient-derived MRD samples, which show the phenotypic heterogeneity of residual leukemic populations, AML cell lines represent clonal populations with fixed phenotypic characteristics and may acquire atypical patterns of surface marker expression. The observed separation can also reflect the consequences of long-term in vitro adaptation. Nevertheless, phenotypic distance from MRD samples should be interpreted as an indicator of biological similarity rather than an absolute measure of model suitability and does not independently determine the relevance of a cell line for CAR-T evaluation. 

The expression level of the particular target antigens represents one of the key factors that should be considered when selecting AML cell line models for CAR-T studies. Antigen expression directly affects CAR-T activation and cytotoxic responses and may also influence the degree of T cell activation and exhaustion during prolonged antigen exposure [18]. Therefore, the biological relevance of a cell line cannot be assessed only based on the presence or absence of a target antigen but should also consider its expression level. For example, CLL1 expression in MOLM-13 was relatively low in comparison with other cell lines in our analysis, which agrees with previously published data and may reflect a clinically relevant antigen expression rather than an insufficient model for CAR-T evaluation [34,35]. Such models may be particularly informative for assessing CAR activity under conditions that more closely resemble the antigen levels observed in primary AML samples. Moreover, the range of antigen relative expression observed across the cell lines may provide a useful framework for subsequent CAR-T optimization, including investigation of antigen-density requirements and the effects of different CAR affinities or avidities. The distinct expression patterns of multiple target antigens may also enable comparative evaluation of single- versus dual- or multi-specific CAR strategies, including logic-gated CAR designs, under conditions of antigen heterogeneity. Thus, antigen-high models may be useful for initial proof-of-concept studies, whereas antigen-low or heterogeneous models may be more informative for specific CAR-T optimization questions. While the scoring system identifies broadly suitable AML models, selection of individual cell lines should ultimately be guided by the CAR target and specific experimental question.

At the same time, cell lines with antigen expression substantially higher than that observed in patient-derived blasts should be interpreted with caution. We observed consistently high CD33 expression across all AML cell lines, more closely resembling those of healthy monocytes than patients’ MRD cells. While high antigen expression in the target AML cell line may simplify the demonstration of CAR-T activity in vitro, such a model may not faithfully represent the antigen relative expression observed in primary AML and should therefore be interpreted carefully when extrapolating preclinical efficacy to the clinical setting. We also evaluated the concordance of our experimental observations with published reports by comparing target antigen expression across AML cell lines. In this context, THP-1 is particularly notable, as the literature contains conflicting reports regarding CLL1 expression in this line: some studies describe it as highly expressed [9,35,36,37], whereas another report low levels of CLL1 in THP-1 [38], which is consistent with our observation. A similar pattern was observed for CD123 expression [39]. A possible explanation for this inconsistency is the cell line’s tendency to differentiate as a result of cultivation, which may affect the expression of certain antigens [40]. For CD123-directed CAR-T studies, target expression may affect both CAR-mediated recognition and AML cell behavior in vivo. This consideration is particularly relevant for NSG-SGM3 xenograft mice models [41], where the expression of human cytokines, including IL-3, may influence the expansion of CD123-positive AML cells [42]. Therefore, CD123 expression should be considered when selecting models for translational studies.

A limitation of this study is that Siglec-6, CSF1R, CD70 expression in cell lines was compared with samples from only three newly diagnosed AML samples, as these markers are not included in our routine MRD panel. These exploratory comparisons require validation in larger patient cohorts.

We also observed differences between AML cell lines in the reduction in viable target cell numbers during co-culture with NT cells. Notably, recent work has shown that B cell-directed CAR-T therapy can activate bystander CD8+ T cells that subsequently acquire a tumor-eliminating phenotype, underscoring the risk of over-attributing cytotoxic effects to CAR specificity when background T cell activation is present [36]. We conducted cytotoxicity assays without the involvement of effector CAR-T cells to assess the baseline level of T cell-mediated cytotoxicity. In our tests, most AML cell lines showed a considerable decrease in cell number as early as the second day of co-culture with T cells. Given that activated T cells express FasL, we initially hypothesized that engagement of Fas on AML cell lines by FasL on activated T cells could provide apoptotic trigger and might explain the enhanced background killing observed in some models, as previously reported by other groups [17,43]. However, no such correlation was found. Moreover, several studies have shown that most AML cell lines, despite the presence of Fas expression, are resistant to Fas-mediated apoptosis due to defects in apoptotic signaling [44,45,46]. A similar limitation applies to the TRAIL pathway, which can also be blocked at multiple levels. Accordingly, AML cell lines are often intrinsically resistant to TRAIL-mediated apoptosis and become sensitive only after pharmacologic sensitization [47,48].

Together, the baseline susceptibility of AML cell lines to non-transduced T cells may be influenced, at least in part, by potential alloreactive recognition of HLA-mismatched targets, although additional mechanisms such as Fas/FasL, TRAIL-mediated cytotoxicity may also contribute. HLA downregulation or loss may contribute to immune evasion and reduced immunogenicity of AML cells [49] and should therefore be considered when interpreting in vitro CAR-T responses, particularly in the context of allogeneic T-cell interactions. Nevertheless, highly T cell-sensitive AML cell lines (e.g., U-937 cell line) may overestimate CAR-T activity, as reduced target cell viability may largely reflect intrinsic susceptibility rather than CAR-dependent cytotoxicity. Since the inclusion of non-transduced T cells as a negative control is essential for distinguishing CAR-dependent target cell killing from intrinsic immune-mediated effects, cell lines with low baseline sensitivity to non-transduced T cells, like OCI-AML5, provide a broader experimental window and allow more reliable normalization of CAR-dependent effects. Therefore, resistance to non-specific T cell-mediated cytotoxicity represents an important parameter for selecting robust in vitro models capable of accurately reflecting CAR-driven functional responses.

Although most AML cell lines used in this study were derived from male donors, except for HL-60, reflecting the male predominance in AML epidemiology and cell line availability, this sex imbalance represents a limitation that may contribute to variability [50,51]. However, antigen density and T-cell-mediated killing are likely to have a greater impact on the observed in vitro phenotype.

A limitation of our study is that the in vitro phenotypic and functional characterization of AML cell lines does not show whether these models can reproduce the cellular, metabolic, and microenvironmental barriers that determine durable CAR-T responses in vivo. Notably, several AML CAR-T studies, including work with adapter CAR platforms, have used different cell lines for in vitro characterization and in vivo efficacy testing, highlighting that strong in vitro activity alone may not necessarily identify the most informative models for subsequent in vivo evaluation [18]. We also examined how frequently these AML cell lines are used in published in vitro and in vivo studies [52,53,54,55,56,57,58,59,60,61,62,63,64,65,66,67,68,69,70,71,72,73,74,75,76,77,78,79,80,81,82,83,84,85,86,87,88,89,90,91,92,93,94,95,96,97,98,99,100,101,102,103,104,105,106,107,108]. Notably, one of the commonly used models, U-937, exhibited the highest level of cell death in the presence of non-transduced primary T cells in our experiments. In contrast, OCI-AML5, which showed low sensitivity to T cell-mediated cell death, was among the least frequently used AML models.

Collectively, our findings highlight the importance of selecting AML cell line models based not only on target antigen expression but also on broader biological characteristics affecting CAR-T assay interpretation. Beyond CAR-T cells, the systematic immunophenotypic, antigen relative expression, growth, and T-cell susceptibility profiles generated here may facilitate rational selection of AML cell line models for antibody-based therapies, antibody–drug conjugates, bispecific T-cell engagers, and combination studies. Because the immunophenotypes of these models were evaluated in relation to MRD-associated phenotypes, this resource may help identify preclinical models that more closely reflect clinically relevant antigenic patterns for testing emerging immunotherapies. By integrating these characteristics, investigators may be able to more rationally select AML models for different preclinical applications and improve comparability across studies.

However, the integrated scoring framework was developed primarily based on CLL1 and CD33, selected as clinically relevant AML targets for CAR-T development, and the resulting ranking should therefore be interpreted mainly in the context of these CAR-T targets. Although the framework provides a general approach for AML cell-line prioritization, adaptation to other target antigens would require incorporation of antigen-specific expression measurements and independent validation. In particular, despite their high overall scores, cell lines OCI-AML2 and OCI-AML5 showed considerable differences in the expression of emerging antigens CD70 and Siglec-6, highlighting that the proposed score does not capture the antigenic heterogeneity across other potential CAR-T targets. Further validation using primary AML samples and in vivo models will be required to determine the extent to which these criteria improve the predictive value of preclinical CAR-T evaluation.

## 5. Conclusions

In summary, this study provides the comprehensive characterization of nine AML cell lines in the context of CAR-T research. It was shown that commonly used AML cell lines may not accurately recapitulate the antigenic profile of primary AML blasts and their sensitivity to T cell-mediated killing should be considered when interpreting in vitro studies. Based on the integrated scoring framework and assay-related factors, OCI-AML2, OCI-AML5, MV4-11, and MOLM-13 received the highest overall scores among the models evaluated. These findings may support the preliminary selection of AML cell lines for future preclinical CAR-T research, particularly targeting CD33 and CLL1.

## Figures and Tables

**Table 1:** AML cell line characterization, data acquired from DSMZ database, https://www.cellosaurus.org/ (accessed on 18 August 2026), and https://www.atcc.org/ (accessed on 18 August 2026).

Cell Line	FAB Subtype	Origin	Cytogenetics	Lineage Markers	HLA Profile	Sources
KG-1	M1	59 y.o. male with erythroleukemia that developed into AML (BM *)	human hypodiploid karyotype with 4.5% polyploidy—45(42–47)<2n>X/XY, −4, +8, +8, −12, −17, −20, +2mar, der(5;17)(q10;q10)del(5)(q?11q?13), dup(7)(q12q33), del(7)(q22q35), i(8q)×2, der(8)t(6;8)(p11;q22), der(8)t(8;12)(p11;q13), der(11)t(1;11)(q13–21;p11–p13), der(16)t(?12;16)(?p13;q13/21)	CD3-, CD4(+), CD13+, CD14−, CD15+, CD19−, CD33+, CD34+, cyCD3(+), cyCD68−	HLA-A*30:02; HLA-B*53:01,*78:01; HLA-C*04:01, *16:01, HLA-DR(+)	DSMZ (ACC 14); ATCC (CCL-246); [21,22]
HL-60	M2	36 y.o. female with acute promyelocytic leukemia (PB **)	human flat-moded hypotetraploid karyotype with hypodiploid sideline and 1.5% polyploidy—82–88<4n>XX, −X, −X, −8, −8, −16, −17, −17, +18, +22, +2mar, ins(1;8)(p?31;q24hsr)×2, der(5)t(5;17)(q11;q11)×2, add(6)(q27)×2, der(9)del(9)(p13)t(9;14)(q?22;q?22)×2, der(14)t(9;14)(q?22;q?22)×2, der(16)t(16;17)(q22;q22)×1–2, add(18)(q21)—sideline with: −2, −5, −15, del(11)(q23.1q23.2)—c-myc amplicons present in der(1) and in both markers	CD3−, CD4+, CD13(+), CD14−, CD15+, CD19−, CD33+, CD34−, cyCD3−, cyCD68−	HLA-A*01:01, *24:15; HLA-B*57:01; HLA-C*06:02, HLA-DR(−)	DSMZ (ACC 3); ATCC (CCL-240); [23,24]
Kasumi-1	M2	7 y.o. male with AML (PB)	human hypodiploid karyotype—45<2n>X, −Y, −9, −13, −16, +3mar, t(8;21)(q22;q22), der(9)t(9;?)(p22;?), der(15)t(?9;15)((?q11;?p11)—carries both partners of 8; 21 translocation associated with AML (mainly FAB M2)	CD3−, CD4+, CD13+, CD14−, CD15(+), CD16(+), CD19−, CD33+, CD34+, CD38+, CD71+, cyCD3−, cyCD68(+)	HLA-A*26:02, *26:01; HLA-B*40:06, *48:01; HLA-C*03:03, *08:01, HLA-DR(+)	DSMZ (ACC 220); ATCC (CRL-2724); [22]
OCI-AML2	M4	65 y.o. male with AML (PB)	human hyperdiploid karyotype with 3.8% polyploidy—48(43–49)<2n>XY, +6, +8, der(1)inv(1)(p36q31)t(1;6) (q13;p12), der(2)t(2;17)(p23;q24.1)del(2)(q14.2q36), der(3)t(1;3)(p36;p25), ins(3;2)(q21;q14.2q36), t(5;8)(q11.2;q24), der(6)t(1;6)(q31;p12)t(3;6) (q26;q24), inv(12)(p13.3q13.2), t(13;14)(q32/33;q24.2), der(17)t(2;17)(p23;q24.1)—sideline with +der(5)—carries apparent variant translocations involving several ANLL breakpoints: 1p36, 3q21, 3q26 (megakaryocytic abnormalities), 12p13, 17q24 (FAB-M4)	CD3−, CD4+, CD13(+), CD14(+), CD15+, CD19−, CD33+, CD34−, cyCD68+	HLA-A*02:01, HLA-B*15:01, 18:01; HLA-C*03:03, *07:01, HLA-DR(+)	DSMZ (ACC 99) [22]
OCI-AML5	M4	77 y.o. male with AML (PB)	human hyperdiploid karyotype—48(44–48)<2n>XY, +1, +8, der(1)t(1;19)(p13;p13)	CD3−, CD4−, CD13−, CD14(+), CD15+, CD16−, CD19−, CD33+, CD34+, cyCD68+, cyCD3−	HLA-A*11:01, *32:01; HLA-B*51:01, *57:01; HLA-C*07:01, *14:02; HLA-DR(+)	DSMZ (ACC 247); [22]
THP-1	M5	1 y.o. male with AML (PB)	human near-tetraploid karyotype—94(88–96)<4n>XY/XXY, −Y, +1, +3, +6, +6, −8, −13, −19, −22, −22, +2mar, add(1)(p11), del(1)(q42.2), i(2q), del(6)(p21)×2–4, i(7p), der(9)t(9;11)(p22;q23)i(9)(p10)×2, der(11)t(9;11)(p22;q23)×2, add(12)(q24)×1–2, der(13)t(8;13)(p11;p12), add(?18)(q21)—carries t(9;11) associated with AML M5	CD3−, CD4 +, CD13+, CD15+, CD19−, CD33+, CD34−, cyCD68+,	HLA-A*02:01, *24:02; HLA-B*15:11, *35:01; HLA-C*03:03; HLA-DR(+)	DSMZ (ACC 16); ATCC (TIB-202); [22]
U-937	M5	37 y.o. male with generalized diffuse histiocytic lymphoma (pleural effusion)	human flat-moded hypotriploid karyotype—63(58–69)<3n>XXY, −2, −4, −6, +7, −9, −20, −21, +3mar, t(1;12)(q21;p13), der(5)t(1;5)(p22;q35), add(9)(p22), t(10;11)(p14;q23), i(11q), i(12p), add(16)(q22), add(19)(q13)—carries t(10;11) (seen in AML M5)— t(1;5) resembles variant of t(2;5) (histiocytic lymphoma)	CD3−, CD4+, CD13−, CD14−, CD15+, CD16−, CD19−, CD33+, CD34−, CD54+, cyCD3−, cyCD68−	HLA-A*03:01; HLA-B*18:01, *51:01; HLA-C*01:02, *07:01; HLA-DR(−)	DSMZ (ACC 5); ATCC (CRL-1593.2); [22,25]
MV4-11	M5	10 y.o. male with AML (PB)	human hyperdiploid karyotype—48(46–48)<2n>XY, +8, +18, +19, −21, t(4;11)(q21;q23)	CD4+, CD5−, CD8−, CD10−, CD13−, CD14−, CD15+, CD19−, CD33+, CD34−, CD68+, CD3−	HLA-A*03:01, *68:01; HLA-B*14:02, *18:01; HLA-C*08:02, *15:02; HLA-DR(+)	DSMZ (ACC 102); ATCC (CRL-9591); [22]
MOLM-13	M5a	20 y.o. male with AML (PB)	human hyperdiploid karyotype with 4% polyploidy—51(48–52)<2n>XY, +8, +8, +8, +13, del(8)(p1?p2?), ins(11;9)(q23;p22p23)—resembles published karyotype—sideline with idem, +19—carries occult insertion effecting KMT2A-MLLT3 (MLL-MLLT3; MLL-AF9) fusion	CD3−, CD4+, CD13(+), CD14−, CD15+, CD19−, CD33+, CD34−, cyCD3−, cyCD68+	HLA-A*31:01, *26:01; HLA-B*51:01, *44:03; HLA-C*14:03, *14:02; HLA-DR(−)	DSMZ (ACC 554); [26]

* BM—bone marrow, ** PB—peripheral blood.

**Table 2:** Scoring framework for ranking AML cell lines for in vitro CAR-T studies. Thresholds were selected a priori for exploratory assessment of AML cell lines as target models for in vitro CAR-T studies. Equal weighting was applied to all criteria, with each score indicating whether a predefined criterion was met. Higher scores therefore indicate greater conformity with the specified model-selection criteria rather than universal suitability for all CAR-T applications.

Parameter	Assessment	0 Points	1 Point	2 Points
CD33 coverage	Percentage of antigen-positive cells	<10%	10–50%	>50%
CD33 expression concordance	MFI relative to MRD blast distribution	Outside Q10–Q90	Q10–Q25 or Q75–Q90	Q25–Q75
CLL1 coverage	Percentage of antigen-positive cells	<10%	10–50%	>50%
CLL1 expression concordance	MFI relative to MRD blast distribution	Outside Q10–Q90	Q10–Q25 or Q75–Q90	Q25–Q75
Blast-like phenotype	Euclidean distance to the MRD cluster centroid in PCA space (lower distance = greater similarity)	>Q75	Median-Q75	<Median
Growth stability	Growth following transfer to the culture medium used for cytotoxicity assays	Failure to maintain stable growth	Stable growth with significant changes after medium adaptation	Stable growth without significant changes after medium transfer
Resistance to NT cells	Reduction in target cell number during NT cell co-culture at E:T = 1:2	>20% reduction	≤20% reduction	No reduction or increased target cell numbers

## Data Availability

The original datasets are presented in the article and Supplementary Materials. Further inquiries can be directed to the corresponding author.

## Supplementary Materials

The following supporting information can be downloaded at https://www.mdpi.com/article/10.3390/cells15211964/s1, references [52,53,54,55,56,57,58,59,60,61,62,63,64,65,66,67,68,69,70,71,72,73,74,75,76,77,78,79,80,81,82,83,84,85,86,87,88,89,90,91,92,93,94,95,96,97,98,99,100,101,102,103,104,105,106,107,108] are cited in the Supplementary Materials. Figure S1: Immunophenotypic profile of AML cell lines in comparison with patient-derived blasts and monocytes. (A–I) Violin plots showing the expression of antigens used for measurable residual disease (MRD) detection (CD13, HLA-DR, CD10, CD11b, CD14, CD99, CD45RA, CD34, CD15, respectively) in patient-derived AML blasts (MRD) and monocytes. MFI of AML cell lines are indicated by horizontal lines. The thick hatching indicates the median, whereas the fine hatching indicates the 25th and 75th percentiles. (J–M). Scatter plots showing autofluorescence of THP-1 cell line in the PE channel (J) and HL-60 cell line in the APC channel (K) compared with MOLM-13 cell line (L,M); Figure S2: Flow cytometric analysis of AML cell line–peripheral blood mixtures. AML cell lines were mixed with peripheral blood (PB) from healthy donors at a 20:80 ratio. AML cell lines and cell line–PB mixtures were stained using the MRD monoclonal antibody panel, CLL1, CD33, CD38, CD117, CD123 expressions are shown. AML cell lines, monocytes, and lymphocytes were identified as distinct populations and are shown in red, green, and blue, respectively; Figure S3: Growth curves of AML cell lines under different culture conditions. (A–G) Comparison of AML cell line adaptation following transfer from standard culture medium (IMDM supplemented with 10% FBS) to the media used for cytotoxicity assays (ImmunoCult and ImmunoCult supplemented with IL-2). Cells were counted every 24 h for 168 h (*n* = 3). Area under the curve (AUC) scores were calculated for each biological replicate to compare growth rates of AML cell lines over time. AUC values were then analyzed using two-way ANOVA (Culture Media × Cell Line). The effect of culture media on AUC scores within each cell line was assessed via pairwise comparisons of estimated marginal means (EMMs), with Tukey’s HSD correction applied for multiple comparisons. Data are presented as mean ± SD. * *p* < 0.05; Figure S4: Cytotoxic activity of T cells against AML cell lines and cytokine release assay. (A–G) AML cell lines were co-cultured with primary non-transduced donor T cells at E ratios of 5:1, 1:2, and 1:4. The number of seeded target cells on day 0 was normalized to 100%, and the relative change in target cell number was assessed on days 2 and 5 relative to day 0 (*n* = 3). Area under the curve (AUC) scores were calculated for each biological replicate to compare T-cell mediated sensitivity to cell death of AML cell lines over time. AUC values were then analyzed using a linear mixed-effects model (E:T ratio X Cell line) with Effector cells donor specified as a random intercept. The effect of E:T ratio on AUC scores within each cell line was assessed via pairwise comparisons of estimated marginal means (EMMs), with Tukey’s HSD correction applied for multiple comparisons. * *p* < 0.05, ** *p* < 0.01, *** *p* < 0.001. (H) IFN-γ release following co-culture of AML cell lines with non-transduced T cells. IFN-γ concentrations were measured in supernatants collected after 48 (D2) and 120 h (D5) of co-culture and normalized to the number of T cells per well; values are presented per 10^5^ T cells (*n* = 3). Data are presented as mean ± SD; Table S1. Antibodies used for flow cytometric immunophenotyping; Table S2: Scoring of AML cell lines according to seven predefined criteria; Table S3: Literature-based assessment of in vitro and in vivo use of AML cell lines in CAR-T studies.

## Abbreviations

The following abbreviations are used in this manuscript:

AML	Acute myeloid leukemia
CAR-T	Chimeric antigen receptor T cell
E:T	Effector-to-target ratio
LAIP	Leukemia-associated aberrant immunophenotype
MFI	Median fluorescence intensity
MRD	Minimal residual disease
NT	Non-transduced T cells
PBMC	Peripheral blood mononuclear cells
PB	Peripheral blood
PCA	Principal component analysis
