# Supplementary material for: Systematic Characterization of AML Cell Lines to Support Preclinical In Vitro CAR-T Research
Source: Cells. 2026 Oct 28;15(21):1964. doi: 10.3390/cells15211964 (PMC13761909; doi:10.3390/cells15211964)
Supplement: Supplementary file 1 [file cells-15-01964-s001.zip › Supplementary tables - revised_28 Oct.pdf]

Table S1. Antibodies used for flow cytometric immunophenotyping

Marker	Fluorochrome	Clone	Manufacturer	Catalog number
MRD panel				
CD15	FITC	MMA	Becton Dickinson	332778
CD34	PE/Dazzle 594	581	BioLegend	343534
CD117	PC5.5	104D2D1	Beckman Coulter	A66333
CD33	PC7	D3HL60.251	Beckman Coulter	A54824
CD13	APC	WM15	BioLegend	301706
CD14	APC-Alexa Fluor 700	RMO52	Beckman Coulter	A99020
CD11b	APC-Cy7	ICRF44	Becton Dickinson	557754
HLA-DR	BV421	L243	BioLegend	307636
CD45	BV510	2D1	BioLegend	368526
CD16	BV605	3G8	Becton Dickinson	563172
CD38	FITC	HIT2	BioLegend	303504
CD371	PE	50C1	BioLegend	353604
CD99	APC	3B2/TA8	BioLegend	371308
CD123	APC-Alexa Fluor 700	SSDCLY107D2	Beckman Coulter	B24028
CD45RA	APC-Alexa Fluor 750	2H4LDH11LDB9	Beckman Coulter	A86050
CD10	BV605	HI10a	Becton Dickinson	562978
CD11a	PE	25.3	Beckman Coulter	IM1433U
CD7	PE	8H8.1	Beckman Coulter	IM1429U
CD2	PE	RPA-2.10	BioLegend	300208
CD4	PE	SK3	BioLegend	344606
CD5	PE	L17F12	BioLegend	364014
CD56	PE	NCAM16.2	Becton Dickinson	345812
CD19	PE	SJ25C1	BioLegend	363004
Additional markers				
CD70	FITC	113-16	BioLegend	355106
CD327	PE	REA852	Miltenyi Biotec	130-112-710
CD115	APC	9-4D2-1E4	BioLegend	347306

Abbreviations: APC, allophycocyanin; BV, Brilliant Violet; FITC, fluorescein isothiocyanate; MRD, measurable residual disease; PE, phycoerythrin; PC5.5, phycoerythrin-cyanine 5.5; PC7, phycoerythrin-cyanine 7.

Table S2. Scoring of AML cell lines according to seven predefined criteria

Cell line	CD33 coverage	CD33 density concordance	CLL1 coverage	CLL1 density concordance	Blast-like phenotype	Growth stability	T-cell resistance	Total score
KG-1	2	1	2	2	0	0	nd	7*
HL-60	2	0	2	0	0	2	nd	6*
Kasumi-1	2	1	1	2	1	1	1	9
OCI-AML2	2	1	2	1	2	2	2	12
OCI-AML5	2	0	2	1	2	2	2	11
THP-1	2	0	0	2	2	2	2	10**
U-937	2	1	2	1	2	1	0	9
MV4-11	2	0	2	2	2	2	2	12
MOLM-13	2	0	2	1	1	2	2	10

nd – not defined

(*) total score was evaluated based on a subset of criteria

(**) despite a high overall score, this cell line was excluded from the final selection because of pronounced autofluorescence, which may interfere with fluorescence-based readouts *in vitro*

Scoring criteria are described in the Results section. Each criterion was scored from 0 to 2, with higher scores indicating greater suitability as an *in vitro* model for CAR-T studies.

Table S3. Literature-based assessment of *in vitro* and *in vivo* use of AML cell lines in CAR-T studies

Cell line	***In vitro* studies, n**	***In vivo* studies, n**	Supporting references
KG-1	8	3	*In vitro:*<br>[10,31,52-57]<br>*In vivo:*<br>[52,56,57]
HL-60	21	6	*In vitro:*<br>[9,11,18,34,37,53-69]

			In vivo:<br>[34,37,56,61,65,66]
Kasumi-1	9	2	*In vitro:*<br>[14,57,64,70-75]<br>*In vivo:*<br>[72,76]
OCI-AML2	8	7	*In vitro:*<br>[13,64,66,75,77-80]<br>*In vivo:*<br>[13,18,64,77-80]
OCI-AML5	1	0	*In vitro:*<br>[81]
THP-1	26	9	*In vitro:*<br>[9,10,30,37,38,54,59,62,64,68,74,75,79,80,82-93]<br>*In vivo:*<br>[74,79,80,82,85,86,90-92]
U-937	17	9	*In vitro:*<br>[11,14,38,54,59,65,68,70,72,75,83,84,93-97]<br>*In vivo:*<br>[14,38,65,68,93-96,98]
MV4-11	15	5	*In vitro:*<br>[11,12,14,18,59,67,75,82,89-92,99-101]<br>*In vivo:*<br>[59,90-92,102]
MOLM-13	30	18	*In vitro:*

			[9-14,31,34,38,56,57,62,66-68,70,75,86,89,93,94,99-101,103-108]<br>*In vivo:*<br>[9-13,31,56,67,70,75,86,99,100,103-107]
